# Endogenous IQGAP1 and IQGAP3 do not functionally interact with Ras

**DOI:** 10.1038/s41598-019-46677-9

**Published:** 2019-07-30

**Authors:** Chase J. Morgan, Andrew C. Hedman, Zhigang Li, David B. Sacks

**Affiliations:** From the Department of Laboratory Medicine, National Institutes of Health, 10 Center Drive, Bethesda, Maryland 20892 USA

**Keywords:** Biological sciences, Oncology, GTP-binding protein regulators

## Abstract

The Ras family of small GTPases modulates numerous essential processes. Activating Ras mutations result in hyper-activation of selected signaling cascades, which leads to human diseases. The high frequency of Ras mutations in human malignant neoplasms has led to Ras being a desirable chemotherapeutic target. The IQGAP family of scaffold proteins binds to and regulates multiple signaling molecules, including the Rho family GTPases Rac1 and Cdc42. There are conflicting data in the published literature regarding interactions between IQGAP and Ras proteins. Initial reports showed no binding, but subsequent studies claim associations of IQGAP1 and IQGAP3 with K-Ras and H-Ras, respectively. Therefore, we set out to resolve this controversy. Here we demonstrate that neither endogenous IQGAP1 nor endogenous IQGAP3 binds to the major Ras isoforms, namely H-, K-, and N-Ras. Importantly, Ras activation by epidermal growth factor is not altered when IQGAP1 or IQGAP3 proteins are depleted from cells. These data strongly suggest that IQGAP proteins are not functional interactors of H-, K-, or N-Ras and challenge the rationale for targeting the interaction of Ras with IQGAP for the development of therapeutic agents.

## Introduction

The Ras superfamily of small GTPases contains >150 proteins in humans^1^. These are grouped into 5 families based on sequence and functional differences. The Ras family, which includes the isoforms H-, K- and N-Ras^1^, controls cell proliferation, survival and cell differentiation. All three Ras isoforms are expressed in all cell types and have very similar (~85% identity) amino acid sequences and structures, with the major variability at their C-tails^2^. The Ras GTPases transduce extracellular signals, notably through the mitogen-activated protein kinase (MAPK) and the phosphoinositide-3-kinase (PI3K)/AKT pathways^3^. A second family, the Rho GTPases, regulates actin dynamics, thereby influencing cell division, cell migration and vesicular transport^1^. Cdc42, Rac1 and RhoA are the best characterized members of the Rho family.

Ras proteins are active when bound to GTP and become inactive when GTP is hydrolyzed to GDP. Activation is regulated by guanine nucleotide exchange factors (GEFs) that reduce the affinity of Ras for GDP, enabling GTP to bind. Inactivation is effected by GTPase activating proteins (GAPs) which stimulate the intrinsic GTPase activity of Ras proteins^1^. Ras activity must be carefully regulated as activating mutations occur in up to 30 percent of human malignancies^4^. Moreover, K-Ras mutations are found in up to 95 percent of pancreatic adenocarcinomas^2^. Thus, inhibition of Ras is a key area of therapeutic research.

The IQ-motif containing Ras GTPase-activating-like protein (IQGAP) family of proteins consists of 3 members in mammals, termed IQGAP1, IQGAP2, and IQGAP3^5^. IQGAPs function as scaffolds for various signaling pathways and modulate diverse biological processes^6^. IQGAP1 is the most well studied member. It is ubiquitously expressed, interacts with over 120 proteins within the cell, and is overexpressed in many different cancers^5,7^. Much less is known about IQGAP2 and IQGAP3. IQGAP2 expression is mainly restricted to the liver, where it appears to have tumor suppressor functions^8^. Little is known about IQGAP3 expression in normal tissues, but like IQGAP1, it is increased in some cancers^9,10^.

Despite their name, IQGAPs do not function as GAPs^11,12^. Nevertheless, they do interact with selected small GTPases. Initial studies of IQGAP1, IQGAP2 and IQGAP3 found that all associated with the GTP-bound forms of Rac1 and Cdc42^11–14^. Further, IQGAP1 and IQGAP2 were shown to inhibit the intrinsic GTPase activity of Rac1 and Cdc42, keeping these proteins in the active state^11–13^. In contrast, all of the early investigations detected no binding of any of the IQGAP proteins to Ras^11,13–16^. Subsequently, cross-linking immunoprecipitation found IQGAP1 as one of many proteins that associate with overexpressed active M-Ras^17^. Neither direct binding nor M-Ras function was examined. More recently, IQGAP1 was reported to interact with K-Ras, but not H-Ras or N-Ras^18^. In addition, IQGAP3 was stated to associate with and alter the activation of H-Ras^19^. The reported interactions of IQGAP1 and IQGAP3 with Ras has generated considerable interest in the research community. Several publications^20–26^, and even a US patent^27^ for cancer immunotherapy, have cited these positive results without acknowledging the earlier negative binding data.

To resolve these discrepant published data, we performed careful and thorough binding analyses. Based on the reported associations^18,19^, we initially hypothesized that IQGAP1 and IQGAP3 could act as isoform-specific Ras scaffolds, coordinating the interaction of different Ras proteins with different effectors. However, we were unable to detect any interaction between H-, K- or N-Ras and endogenous IQGAP1 or IQGAP3 by immunoprecipitation or pull down assays, despite using high levels of the active Ras isoforms. Importantly, knockdown of IQGAP1 or IQGAP3 had no significant effect on activation of Ras in HeLa cells. Our results do not support a functional interaction between Ras and IQGAP1 or IQGAP3.

## Results

### Validation of a polyclonal anti-IQGAP3 antibody

**Figure 1:**
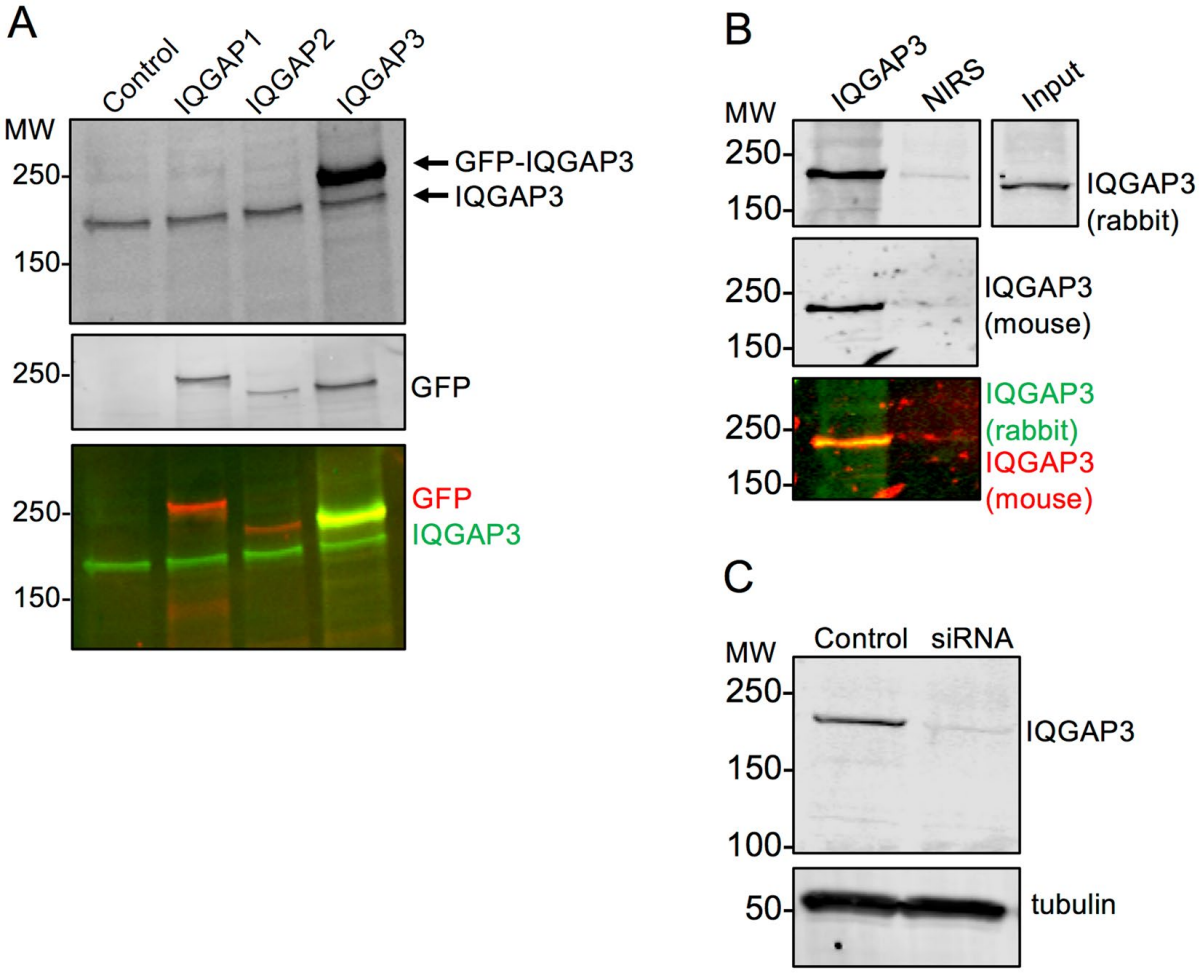
Anti-IQGAP3 Rabbit Polyclonal Antibody Specifically Detects IQGAP3. (**A**) HEK293 cells were transfected with GFP-IQGAP1, GFP-IQGAP2, GFP-IQGAP3, or not transfected (control). Equal amounts of cell lysate were analyzed by SDS-PAGE and Western blotting. Blots were probed sequentially with anti-IQGAP3 rabbit polyclonal antibody (black in top panel, green in bottom panel) and anti-GFP mouse monoclonal antibody (black in middle panel, red in bottom panel). Yellow indicates co-localization. Panels were derived from the same blot. (**B**) Endogenous IQGAP3 was immunoprecipitated from HEK293 cells with anti-IQGAP3 rabbit polyclonal antibody. Non-immune rabbit serum (NIRS) was used as a negative control. Both unfractionated lysate (Input) and complexes were resolved by SDS-PAGE and Western blotting. The blot was probed sequentially with anti-IQGAP3 rabbit polyclonal antibody (black in top panel, green in bottom panel) and a commercially available anti-IQGAP3 mouse polyclonal antibody (black in middle panel, red in bottom panel). Yellow indicates co-localization. Panels were derived from the same blot. (**C**) A549 cells were transfected with IQGAP3 targeted siRNA. Lysates from untransfected (control) and siRNA transfected cells were analyzed by SDS-PAGE and Western blotting. Blots were probed with anti-IQGAP3 rabbit polyclonal antibody and anti-tubulin antibody (loading control). Panels were cropped from the same blot. The positions of migration of molecular weight markers are indicated on the left. Blots for all panels were cropped for clarity. The full-length blots can be found in Supplementary Fig. 1.

In order to perform these studies, we initially developed and validated an anti-IQGAP3 rabbit polyclonal antibody. The antibody was developed as described in Experimental Procedures. HEK293 cells were transfected with GFP-tagged IQGAP1, IQGAP2, or IQGAP3 plasmids, lysed, and analyzed by SDS-PAGE and Western blotting. Blots were probed with both the anti-IQGAP3 rabbit polyclonal antibody and an anti-GFP mouse monoclonal antibody. The data show that the anti-IQGAP3 antibody recognizes both endogenous and GFP-tagged IQGAP3 (Fig. 1A). Importantly, the antibody does not cross-react with GFP-IQGAP1 or GFP-IQGAP2. Probing the blot with anti-GFP antibody shows the expression of GFP-tagged IQGAP1, IQGAP2, and IQGAP3 (Fig. 1A, middle panel). Endogenous IQGAP3 was also immunoprecipitated from cell lysates with the anti-IQGAP3 rabbit polyclonal antibody. Western blots were probed both with the same anti-IQGAP3 rabbit polyclonal antibody (Fig. 1B, top panel) and with a commercially available anti-IQGAP3 mouse polyclonal antibody (Fig. 1B, middle panel). These data reveal that both antibodies recognize the same band. Minimal IQGAP3 was present in the sample immunoprecipitated with non-immune rabbit serum (Fig. 1B). Lastly, A549 cells were transfected with IQGAP3-specific siRNA and the level of IQGAP3 protein was compared to that in untransfected cells. Probing the Western blot with our anti-IQGAP3 rabbit polyclonal antibody revealed a marked reduction in IQGAP3 protein in the siRNA transfected sample (Fig. 1C). Collectively, these experiments demonstrate that the anti-IQGAP3 antibody we developed specifically recognizes IQGAP3.

### Protein-protein interaction databases show low likelihood of IQGAP-Ras interactions

Protein-protein interaction databases were searched for evidence of binding between IQGAP and Ras (Table 1). BioGRID is a database that compiles interactions recorded in the literature^28^. Despite data from multiple high-throughput studies for IQGAP1, IQGAP2, and IQGAP3 binding partners, there are no recorded interactions with Ras in BioGrid. IntAct is a database that compiles interactions based upon literature curation and user submission. Again, no binding events between Ras and either IQGAP1, IQGAP2, or IQGAP3 were recorded. STRING, another commonly used protein interaction database that compiles interactions from the literature^29^, was omitted from this table due to a clear error in its algorithm. STRING stated that all Ras isoforms were IQGAP1 binding partners. However, the only manuscript cited to support this claim^18^ specifically stated that neither N-Ras nor H-Ras binds to IQGAP1. The PrePPI database assigns likelihood ratios for predicted protein-protein interactions based upon structural, functional, evolutionary, and expression information, as well as interactions recorded in the published literature^30^. A likelihood ratio cutoff of 0.5 is used to determine high confidence interactors. The likelihood ratio for the interaction of IQGAP1 with K-Ras or N-Ras is 0.53, while that for H-Ras is below the cutoff. The likelihood ratio for IQGAP3 and K-Ras interaction is 0.5, while the other two Ras isoforms fall below the threshold. The interaction likelihood ratio for all Ras isoforms was below the 0.5 cutoff for IQGAP2. In comparison, the likelihood ratios for the interactions of Cdc42 with IQGAP1, IQGAP2, and IQGAP3 are all 1. Together, the results of these searches suggest a low likelihood that any isoform of Ras binds to IQGAP.

**Table 1:** Protein-Protein Interaction Databases Do Not Show Evidence of a Ras-IQGAP Interaction.

Database	Type of Database	Probability of Ras Interaction with IQGAP1	Probability of Ras Interaction with IQGAP2	Probability of Ras Interaction with IQGAP3
BioGrid	Experimental	No Interaction	No Interaction	No Interaction
IntAct	Experimental	No Interaction	No Interaction	No Interaction
PrePPI	Experimental/Prediction	0.53 (K-Ras, N-Ras)	<0.5	0.5 (K-Ras only)

### Neither endogenous IQGAP1 nor IQGAP3 co-immunoprecipitates with Ras

**Figure 2:**
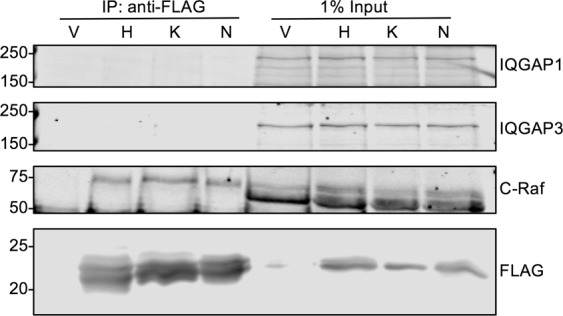
Neither Endogenous IQGAP1 nor IQGAP3 Co-Immunoprecipitates with H-, K-, or N-Ras. HEK293 cells were transfected with FLAG-tagged, constitutively active (G12V) H-, K-, or N-Ras or vector alone (V). Cells were lysed and FLAG was immunoprecipitated (IP) with anti-FLAG antibody. Equal amounts of lysate were loaded directly onto the gel (1% input). Samples were resolved by SDS-PAGE and Western blotting. Blots were probed with anti-IQGAP1, anti-IQGAP3, anti-C-Raf, and anti-FLAG antibodies. The panels depict different sections and different exposures of the same blot. The full-length blot can be found in Supplementary Fig. 2. Data are representative of five independent experiments.

There are no reported interactions between IQGAP2 and Ras. Moreover, as IQGAP2 appears to be a tumor suppressor, rather than an oncogene^6^, we decided not to evaluate IQGAP2 in this study. To examine the possible interaction of IQGAP1 and/or IQGAP3 with Ras in cells, we initially performed immunoprecipitation. HEK293 cells, which contain both IQGAP1 and IQGAP3 (Fig. 2), were used. Cells were transfected with FLAG-tagged constitutively active (G12V) H-Ras, K-Ras, or N-Ras constructs. Cells were then lysed and the transfected Ras constructs were immunoprecipitated with an anti-FLAG antibody. Immunoprecipitates were analyzed by SDS-PAGE and Western blotting. No IQGAP1 was detected in immunoprecipitates of any of the three Ras isoforms, H-Ras, K-Ras, or N-Ras (Fig. 2). Similarly, IQGAP3 was not detectable in Ras immunoprecipitates. To determine whether the Ras proteins were capable of binding endogenous protein partners, the blots were probed for C-Raf. As anticipated, C-Raf co-immunoprecipitated with all three Ras isoforms (Fig. 2). Probing the blots with anti-FLAG antibody shows that approximately equal amounts of each Ras isoform were expressed in the cells and were immunoprecipitated. Immunoprecipitates from cells transfected with vector only demonstrate specificity. Western blots of cell lysates reveal that equal amounts of protein were present in each sample (Fig. 2). These data reveal that, despite overexpression, neither H-Ras, K-Ras, nor N-Ras is in a complex with endogenous IQGAP1 or IQGAP3 in cell lysates.

### Neither endogenous IQGAP1 nor IQGAP3 is detectable in GST-Ras pull downs

**Figure 3:**
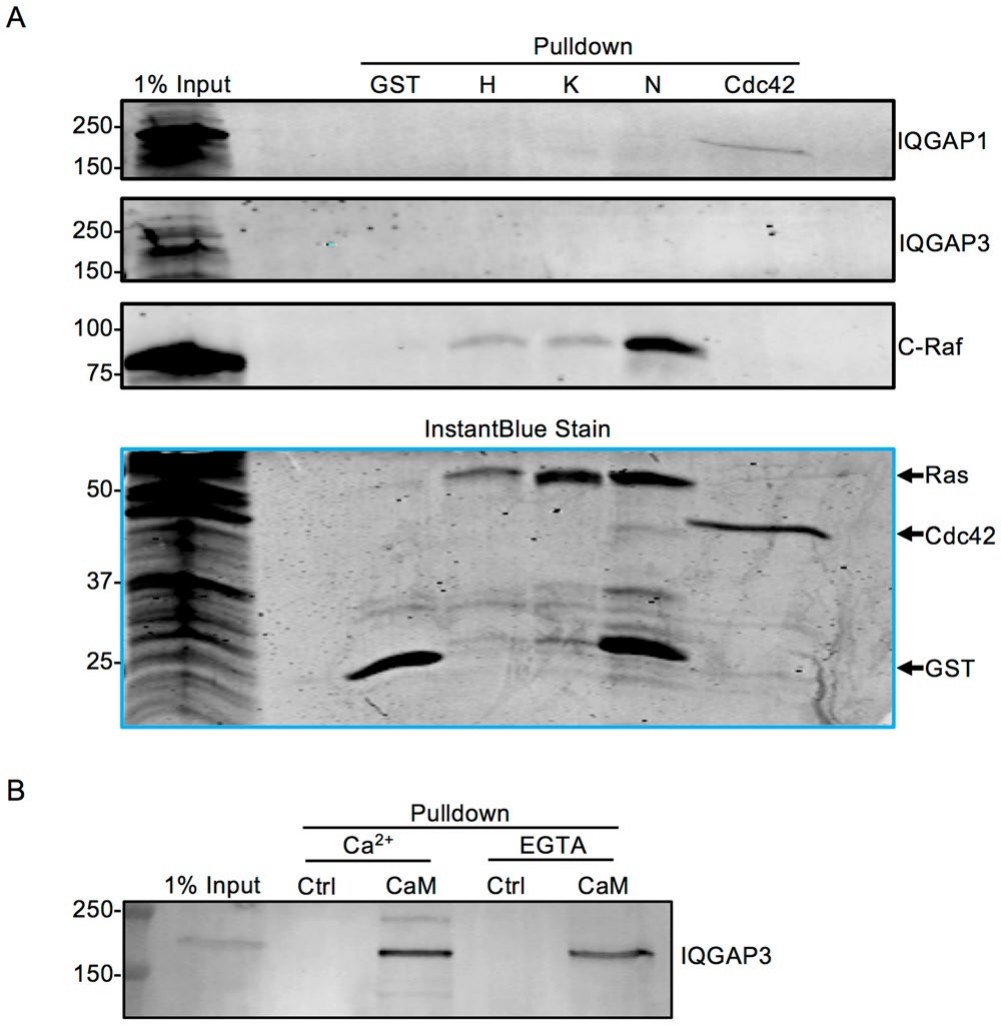
Neither Endogenous IQGAP1 nor IQGAP3 is Detectable in GST-Ras Pull Downs. (**A**) HEK293 cells were transfected with Myc-C-Raf and lysed. Lysates were incubated with equal amounts of GST-tagged constitutively active (G12V) H-, K-, or N-Ras, GST-tagged constitutively active (Q61L) Cdc42, or GST protein. 1% of lysate subjected to pull down was loaded directly onto the gel (1% input). Samples were resolved by SDS-PAGE and gels were cut at ~60 kDa. The upper portions of the gels were transferred to PVDF and probed with anti-IQGAP1, anti-IQGAP3, and anti-C-Raf antibodies. The top three panels were derived from the same blot. The lower portions of the gels were incubated with InstantBlue protein stain. Data are representative of four independent experiments. (**B**) Equal amounts of HEK293 cell lysates were incubated with calmodulin-Sepharose (CaM) or glutathione-Sepharose (negative control; Ctrl) in the presence of 1 mM CaCl_2_ or 1 mM EGTA. Bound proteins were analyzed by SDS-PAGE and Western blotting. 1% of the lysate was loaded directly onto the gel (1% input). The panel depicts a cropped section of a single blot. Data are representative of two independent experiments. The positions of migration of molecular weight markers are indicated on the left. The full-length blots and gel can be found in Supplementary Fig. 3.

Next, we attempted to identify an interaction with IQGAP by pull down using purified recombinant GST-Ras G12V proteins expressed in *E. coli*. GST-tagged H-, K-, or N-Ras was incubated with HEK293 lysates. After pulldown with glutathione-Sepharose beads, bound proteins were analyzed by SDS-PAGE and Western blotting. Neither endogenous IQGAP1 nor endogenous IQGAP3 could be detected in pull downs with any of the three GST-Ras isoforms (Fig. 3A). In contrast, C-Raf bound to each of the Ras isoforms, confirming that the constructs are capable of interacting with at least one Ras binding partner. While more C-Raf is detected in the N-Ras pull down in Fig. 3A, this was an isolated finding and was not observed consistently. Pull down with GST-Cdc42 confirmed endogenous IQGAP1 binding to a GST-tagged protein (Fig. 3A). Unexpectedly, endogenous IQGAP3 was not detected in the GST-Cdc42 pull down. As anticipated, C-Raf did not bind GST-Cdc42 (Fig. 3A). The InstantBlue stain shows that approximately equal amounts of GST-tagged proteins were present in each condition. These results are in agreement with our immunoprecipitation data and further demonstrate that Ras does not form a complex with either endogenous IQGAP1 or endogenous IQGAP3.

### Endogenous IQGAP3 is precipitated by calmodulin

In order to determine if endogenous IQGAP3 can be detected in pull down experiments, we examined calmodulin binding. Calmodulin binds IQGAP1^31^ and IQGAP2^13^. Like the other IQGAP proteins, IQGAP3 contains four IQ motifs^6^, the region to which calmodulin binds. However, there are no published data that document binding of calmodulin to full-length IQGAP3. We incubated HEK293 cell lysates with calmodulin-Sepharose. Endogenous IQGAP3 bound to calmodulin in the presence of Ca^2+^ (Fig. 3B). When free Ca^2+^ was chelated with EGTA, IQGAP3 still bound calmodulin, but the amount was reduced by 20%. No IQGAP3 was detected in samples pulled down with control Sepharose beads, validating binding specificity (Fig. 3B). Collectively, Our data reveal that endogenous IQGAP3 binds calmodulin.

### Overexpressed IQGAP1 and IQGAP3 can be detected in GST-Ras pull downs

**Figure 4:**
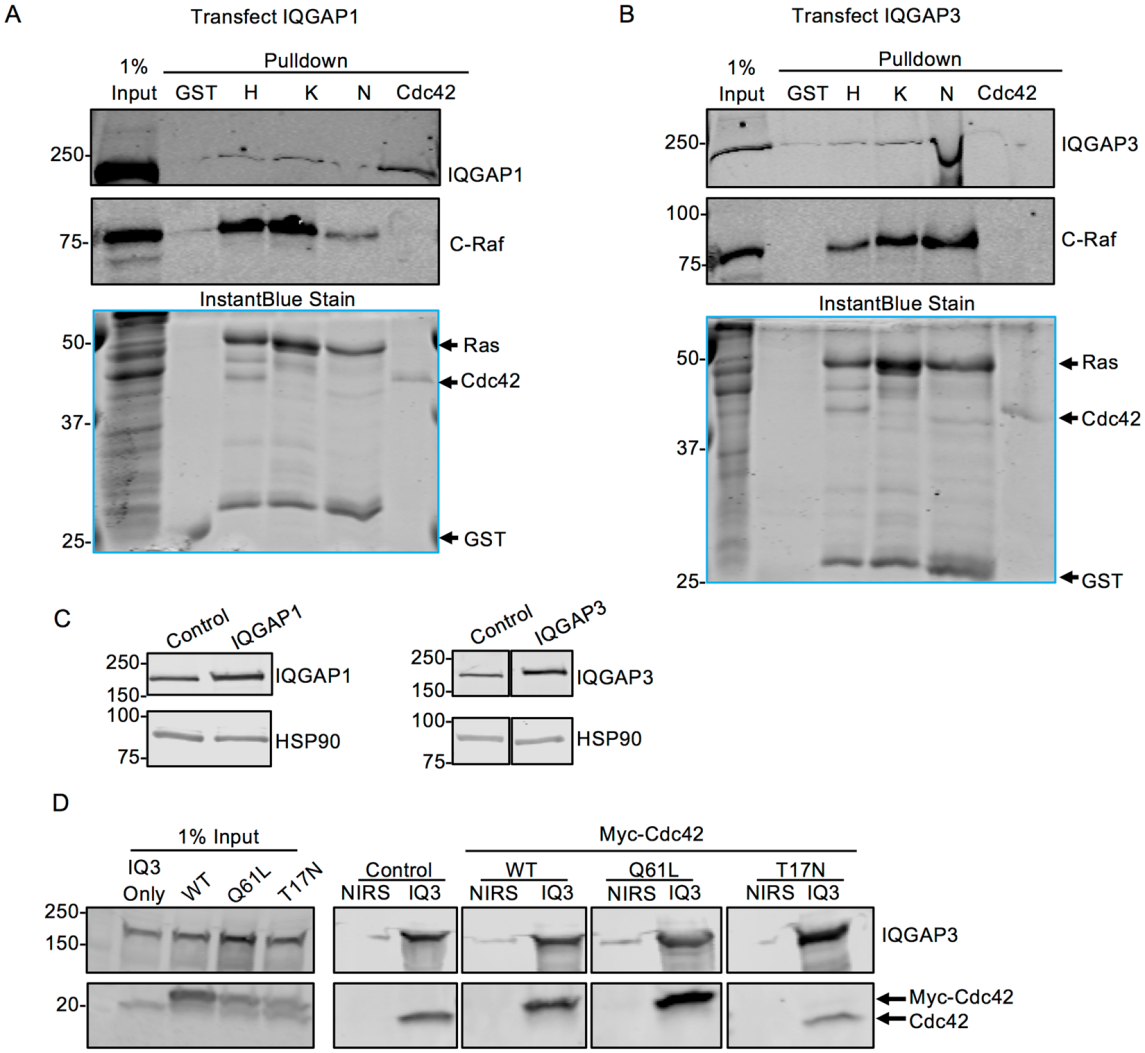
Overexpressed IQGAP1 and IQGAP3 Can Be Detected in GST-Ras Pull Downs. (**A**) HEK293 cells were transfected with both Myc-C-Raf and Myc-IQGAP1 and lysed. Lysates were incubated with equal amounts of GST-tagged constitutively active (G12V) H-, K-, or N-Ras, GST-tagged constitutively active (Q61L) Cdc42, or GST protein. 1% of the lysate was loaded directly onto the gel (1% input). Samples were resolved by SDS-PAGE and gels were cut at ~60 kDa. The upper portion of the gels was transferred to PVDF and probed with anti-IQGAP1 and anti-C-Raf antibodies. The Top two panels depict different sections of the same blot. The lower portion of the gels was incubated with InstantBlue protein stain. (**B**) HEK293 cells were transfected with both Myc-C-Raf and Myc-IQGAP3. Samples were processed as described for A, except blots were probed with anti-IQGAP3 antibody. (**C**) Lysates from HEK293 cells untransfected (control) or transfected with Myc-IQGAP1 or Myc-IQGAP3 were analyzed by SDS-PAGE and Western blotting. Blots were probed for IQGAP1 (left panel) or IQGAP3 (right panel) and HSP90 (loading control). The left panels were derived from the same blot. The right panels were derived from a single blot. (**D**) HEK293 cells were transfected with Myc-IQGAP3. Where indicated, cells were also transfected with wild type (WT), constitutively active (Q61L), or dominant negative (T17N) Myc-Cdc42. Cells were lysed and IQGAP3 was immunoprecipitated with anti-IQGAP3 antibody (IQ3). Non-immune rabbit serum (NIRS) was used as a negative control. 1% of the lysate was loaded directly onto the gel (Input). Samples were analyzed by SDS-PAGE and Western blotting. Blots were probed with anti-IQGAP3 and anti-Cdc42 antibodies. Endogenous Cdc42 and transfected Myc-Cdc42 are distinguished by their difference in migration. The left panels were derived from a single blot. The other four panels were derived from a single blot. The positions of migration markers are indicated on the left. Data are representative of three independent experiments (Panels A, B, C) or five independent experiments (Panel D). Blots were cropped for clarity. The full-length blots and gels can be found in Supplementary Fig. 4.

The only studies that have reported interactions between Ras and IQGAP1 or IQGAP3 used cells overexpressing both proteins^18,19^. Therefore, we transfected HEK293 cells with either Myc-IQGAP1 or Myc-IQGAP3 and incubated the cell lysates with GST-Ras. Under these conditions, a faint IQGAP1 band could be detected in pull downs with all three Ras isoforms (Fig. 4A). Similarly, IQGAP3 was detectable in the Ras pull downs (Fig. 4B). IQGAP1 bound to GST-Cdc42, but minimal IQGAP3 is visible. C-Raf bound all Ras isoforms, but not Cdc42 (Fig. 4A,B). Minimal IQGAP1, IQGAP3, or C-Raf was detected in pull downs with GST alone. The InstantBlue stain demonstrates that approximately equal amounts of GST-tagged proteins were present in each sample (Fig. 4A,B). Note that transfection increased the expression of IQGAP1 or IQGAP3 two-fold over endogenous levels (Fig. 4C). Quantification of several Western blots revealed that transfected cells expressed two-fold more IQGAP than control cells.

### Cdc42 co-immunoprecipitates with IQGAP3

To investigate the unexpected failure of IQGAP3 to bind Cdc42, immunoprecipitation experiments were performed with wild type, constitutively active (Q61L), and dominant negative (T17N) Cdc42. HEK293 cells were transfected with IQGAP3 alone or IQGAP3 plus each Cdc42 construct. IQGAP3 was immunoprecipitated and complexes were analyzed by SDS-PAGE and Western blotting. Both endogenous Cdc42 and transfected wild type Cdc42 co-immunoprecipitated with IQGAP3 (Fig. 4D). As anticipated, Cdc42 Q61L also co-immunoprecipitated with IQGAP3 and the amount was 1.7-fold greater than wild type Cdc42. In contrast, no Cdc42 T17N bound to IQGAP3 (Fig. 4D). Comparable amounts of IQGAP3 are present in each immunoprecipitate. These results indicate that IQGAP3 interacts with Cdc42 in cell lysates. To our knowledge, this is the first time that IQGAP3 has been shown to interact with endogenous Cdc42.

### Endogenous calmodulin and Cdc42, but not endogenous Ras, associate with endogenous IQGAP1 and IQGAP3

**Figure 5:**
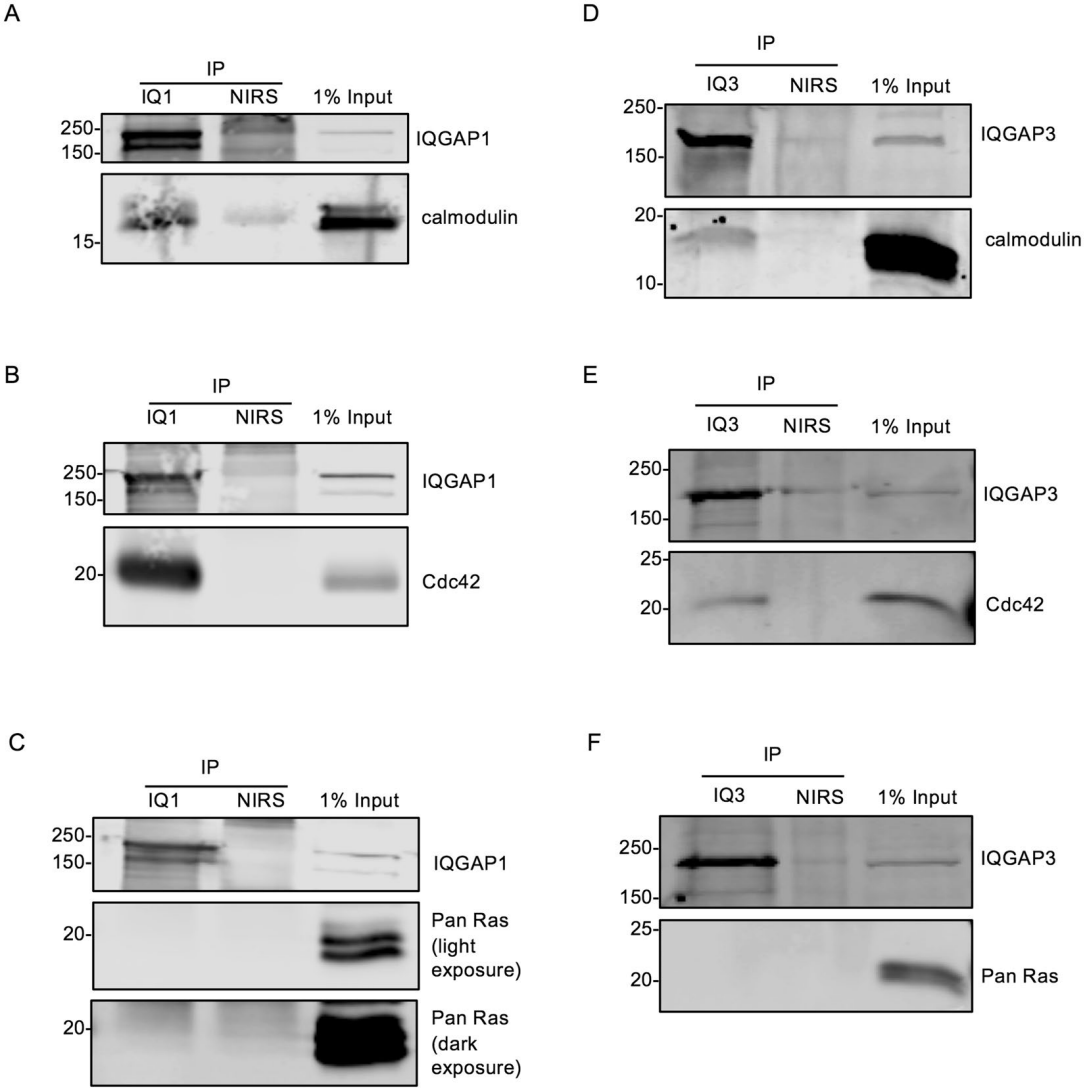
Endogenous Calmodulin and Cdc42, but not Endogenous Ras, Associate with Endogenous IQGAP3 and IQGAP1. Endogenous IQGAP1 (IQ1) (panels A, B and C) or IQGAP3 (IQ3) (panels D, E and F) was immunoprecipitated (IP) from untransfected HEK293 cell lysates. Non-immune rabbit serum (NIRS) was used as a negative control. Precipitates and 1% of the lysate (1% input) used for each experiment were resolved by SDS-PAGE and analyzed by Western blotting. Blots were probed for IQGAP1 (panels A, B and C) or IQGAP3 (panels D, E and F) and calmodulin (panels A, D), Cdc42 (panels B,E), or Ras (panels C, F). Ras was detected using a pan Ras antibody that recognizes the isoforms H-,K-, and N-Ras. Numbers indicate the positions of migration of molecular weight markers. Each panel depicts two sections from a single blot (i.e. three blots in total for IQGAP1 and three for IQGAP3). Blots were cropped for clarity. The full-length blots can be found in Supplementary Fig. 5. Data shown are representative of at least two independent experiments.

Since all the preceding experiments were performed with at least one protein in excess, we immunoprecipitated endogenous IQGAP1 or IQGAP3 from untransfected HEK293 cells to investigate their association with endogenous protein partners. Calmodulin (Fig. 5A) and Cdc42 (Fig. 5B) were detected in IQGAP1 immunoprecipitates. However, no Ras was detectable when blots were probed with a pan Ras antibody that detects the isoforms H-, K-, and N-Ras, even with dark exposure of the Western blot (Fig. 5C). Essentially identical observations were made with IQGAP3; endogenous calmodulin (Fig. 5D) and Cdc42 (Fig. 5E), but not Ras (Fig. 5F), were present in IQGAP3 immunoprecipitates. Non-immune rabbit serum controls demonstrate specificity. These data reveal that Cdc42 and calmodulin are endogenous binding partners of both IQGAP1 and IQGAP3, and provide further evidence that Ras is not an IQGAP1 or IQGAP3 binding partner.

### Knockdown of IQGAP1 or IQGAP3 does not alter Ras activation

**Figure 6:**
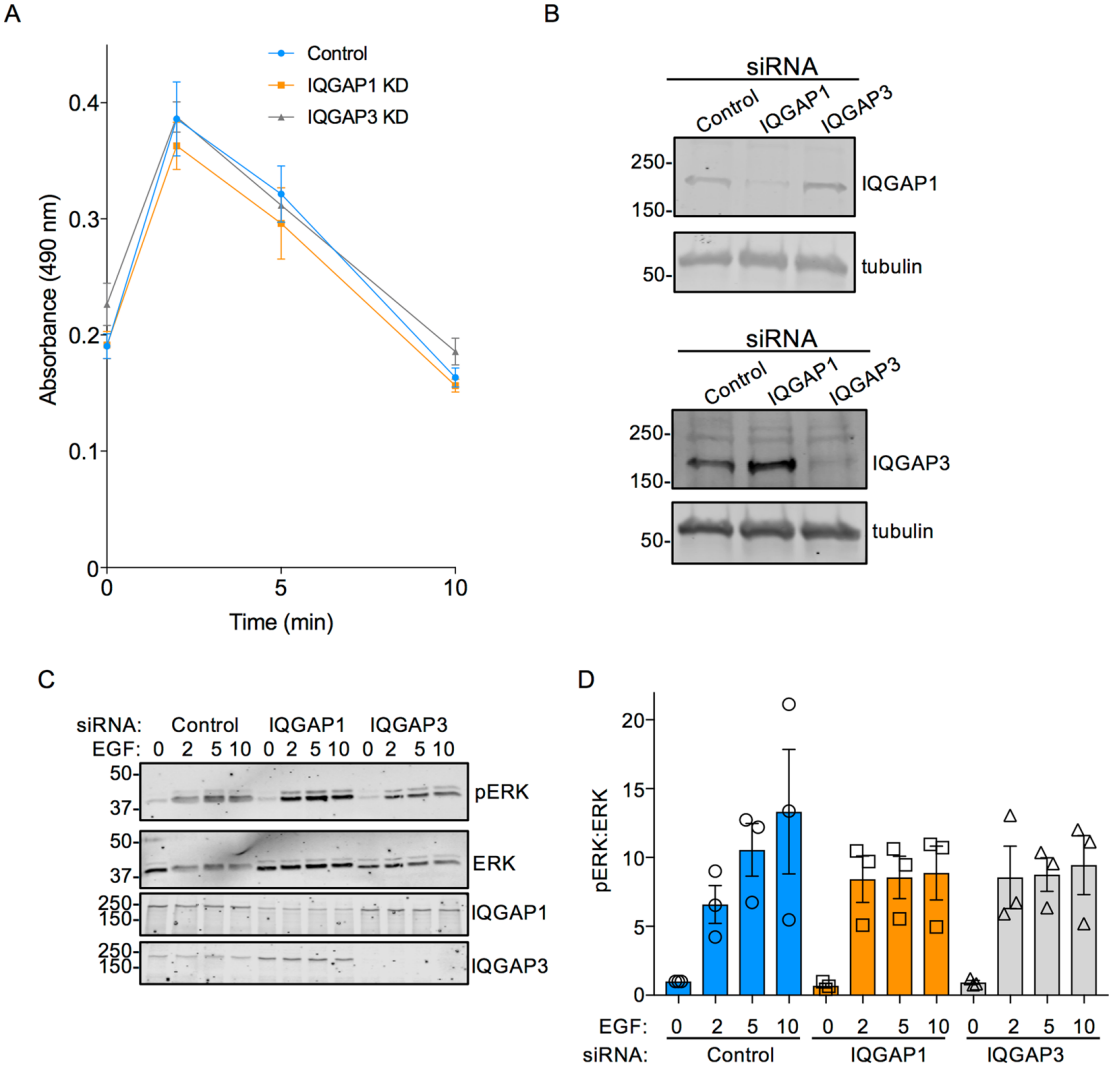
Knockdown of IQGAP1 or IQGAP3 Does Not Alter Ras Activation. HeLa cells were transfected with control, IQGAP1, or IQGAP3 siRNA for 72 h. After overnight serum starvation, cells were stimulated with 100 ng/mL EGF for 0, 2, 5, or 10 min and lysed. (**A**) Equal amounts of protein were analyzed by Ras G-Lisa assay, which quantifies GTP-bound Ras. The graph shows the absorbance at 490 nm. Absorbance is directly proportional to the amount of active GTP-bound Ras in the sample. Data are from two independent experiments, each performed in triplicate and represent means +/− SEM. (**B**) Equal amounts of protein from transfected cells were analyzed by SDS-PAGE and Western blotting. Blots were probed for IQGAP1 or IQGAP3, and tubulin (loading control). The positions of migration of molecular weight markers are indicated. The top two panels are different sections of a single blot. The bottom two panels are different sections of a single blot. The full-length blots can be found in Supplementary Fig. 6. Data are representative of two independent experiments. (**C**) Equal amounts of protein were analyzed by SDS-PAGE and Western blotting and probed with antibodies to phospho-ERK (pERK), total ERK, IQGAP1 and IQGAP3. The blots were cut at ~100 kDa. Blots were cropped for clarity. The full-length blots can be found in Supplementary Fig. 6. (**D**) phospho-ERK was quantified with Image Studio 2.0 (LI-COR) and corrected for the amount total ERK in the same sample. Data are expressed as means ± S.E.M. (n = 3), with untreated control siRNA condition set as 1.

If IQGAP1 and/or IQGAP3 have functional interactions with Ras, one would anticipate that altering the intracellular IQGAP concentration would influence the activation of Ras. To test this, we knocked down IQGAP1 or IQGAP3 in HeLa cells using siRNA. Cells were then incubated with EGF, a known activator of Ras, for different time intervals and active, GTP-bound Ras was quantified. EGF significantly increased the amount of active Ras in control cells in a time-dependent manner (Fig. 6A). Maximal activation occurred at 2 min and returned to baseline by 10 min. Depletion of IQGAP1 or IQGAP3 had no significant effect on either the magnitude or the kinetics of EGF-stimulated Ras activation (Fig. 6A). IQGAP1 knockdown did slightly, but not significantly, decrease the maximal activation of Ras, but did not alter the kinetics. The most likely explanation for this observation is that IQGAP1 knockdown impairs activation of EGF receptors^32^. Western blots confirm reduction in IQGAP1 and IQGAP3 expression (Fig. 6B). These data indicate that neither IQGAP1 nor IQGAP3 influences Ras activation or inactivation after EGF stimulation. Taken together, our data strongly suggest that IQGAP proteins do not have a functional interaction with Ras in cells.

The potential involvement of IQGAP1 and IQGAP3 in MAPK signaling was examined in HeLa cells. IQGAP1 or IQGAP3 was reduced with siRNA and cells were treated with EGF. As anticipated, MAPK signaling (as measured by phosphorylation of ERK1/2) was increased rapidly and substantially in cells transfected with control siRNA (Fig. 6C,D). Reducing IQGAP1 or IQGAP3 attenuated the effect of EGF at the 5 and 10 min time points. Consistent with previous studies^10,33^, knockdown of either IQGAP1 or IQGAP3 did not completely eliminate EGF-stimulated ERK activation, which suggests that there may be redundant roles for IQGAP1 and IQGAP3 in this pathway. Therefore, any future IQGAP-directed therapies may need to target both proteins.

## Discussion

Defective control of Ras activity is linked to numerous human pathologies, including many cancers^4^. Notwithstanding over 35 years of investigation, no effective therapy has been developed for the large number of patients with Ras-mutated malignancies^34^. Directly targeting Ras is difficult as it contains few druggable binding sites. A conceptually appealing approach to inhibit Ras signaling is to disrupt the interaction between Ras and Ras-binding proteins^3^. IQGAP1 and IQGAP3 are overexpressed in neoplasia^7,9^ and have been proposed as oncogenes. IQGAP1 binds myriad proteins, including members of the Ras superfamily^5^. While binding of IQGAPs to selected Rho GTPases is well-characterized and accepted^5^, the published literature contains conflicting findings regarding the interaction of IQGAPs with H-, K- and N-Ras^11,13–16,18,19^. In order to settle the controversy, we set out to carefully evaluate this problem and determine if IQGAP1 and/or IQGAP3 bind to and modulate the levels of active Ras.

In this study, we made three key observations. First, no endogenous IQGAP1 or IQGAP3 associates with constitutively active H-, K-, or N- Ras isoforms. Second, association of IQGAP with Ras could be discerned only when IQGAP was overexpressed. Most importantly, we observed that knockdown of IQGAP1 or IQGAP3 had no effect on EGF-stimulated Ras activation. Altogether, these finding support distinct functions for IQGAP and Ras in MAPK signaling.

To assess a potential association between Ras and IQGAP, we attempted to pull down endogenous IQGAP proteins using immunoprecipitated or purified Ras proteins. Under these conditions, both of which have excess constitutively active Ras, no IQGAP1 or IQGAP3 was detectable, while the established Ras effector C-Raf readily associated with all three Ras isoforms. Similarly, Ras failed to co-immunoprecipitate from cells with endogenous IQGAP1 or IQGAP3, although calmodulin and Cdc42 were clearly present. The two previous publications that reported IQGAP association with Ras used overexpression^18,19^. Therefore, we overexpressed IQGAP1 or IQGAP3 at least two-fold in cells to determine if any association could be detected. Under these overexpression conditions, a small amount of IQGAP1 or IQGAP3 could be discerned in samples pulled down by purified, constitutively active Ras. Note that despite overexpressing GFP-IQGAP3 in Cos-7 cells, Wang *et al*.^14^ observed no IQGAP3 in pull downs with GTPγS-loaded GST-H-Ras. Therefore, it seems that detectable association between IQGAP and Ras is possible only with supra-physiological amounts of both proteins.

Our failure to identify a clear and robust interaction between IQGAP1 or IQGAP3 and Ras concurs with all the initial reports which indicate that the proteins do not bind^11,14,16^. When first identified, IQGAP1 was thought to function as a RasGAP^35^. Notwithstanding this hypothesis, several different research groups were unable to observe binding of IQGAP1, IQGAP2 or IQGAP3 to Ras^11,13–16^. None of these studies, which used recombinant Ras to evaluate interactions with endogenous IQGAP proteins, detected any association under any conditions. All of the initial observations imply that IQGAP proteins are unable to bind Ras isoforms. In addition, we queried several databases that use predictions based on protein structures and amino acid sequences, as well as data from the literature and high throughput studies; all of these databases indicate either a very low probability of binding or no binding between any IQGAP and any Ras isoform (Table 1). Nevertheless, using mass spectrometry one manuscript proposed an association between IQGAP1 and overexpressed constitutively active K-Ras, and indicated that IQGAP1 co-immunoprecipitated with overexpressed active K-Ras, but not active H-Ras or N-Ras^18^. Additionally, one publication reported that IQGAP3 and H-Ras associate, but both IQGAP3 and Ras were overexpressed in all of the association experiments^19^. Neither of the two studies that reported an association accounted for differences between their data and those in prior publications.

Although it is impossible to prove that two proteins never interact under any condition, our binding data, combined with observations in previous publications^11,13–16,18,19^, strongly suggest that IQGAP and Ras association may be perceptible only under conditions where expression of both IQGAP and Ras is enhanced. Non-specific protein-protein interactions may occur when proteins are present at supra-physiological concentrations^36^, such as transient or stable protein overexpression. While it is conceivable that there may be some human malignancies where increased expression of IQGAP may permit interaction with Ras, these anomalous conditions are unlikely to represent major functions of Ras or IQGAP in cells. Furthermore, it appears likely that any interaction of Ras with IQGAP is indirect, via a common binding partner. In support of this hypothesis, there are no published data that document direct binding. Investigations that evaluated binding using pure proteins report that there is no interaction^14^. Over seven distinct Ras effector proteins have been identified in cancer cells^1^. Importantly, IQGAP1 is known to bind to most of these, including B-Raf^37^, C-Raf^38^, PI3K^33^, Tiam1^39^, and PLCε1^40^. Moreover, both K-Ras^41^ and IQGAP1^31^ directly bind calmodulin. IQGAP3 binding partners are less well characterized, but associations of IQGAP3 with B-Raf have been reported^42^ and here we demonstrate that IQGAP3 binds calmodulin (Figs 3B and 5D). Therefore, it seems reasonable to postulate that the IQGAP detected in the pull downs with active Ras is mediated via one or more of these common binding partners, rather than via a direct interaction between Ras and IQGAP. For example, all our Ras pull downs readily precipitated C-Raf, which may be one potential protein that contributes to the presence of IQGAP proteins in Ras pulldowns.

Numerous stimuli activate Ras, which then binds its effectors to propagate signaling to drive cellular processes, including proliferation and migration^2^. The best characterized Ras regulated pathway is the MAPK cascade, where Ras binds and activates Raf, initiating sequential phosphorylation of MEK and ERK^4,43^. Importantly, IQGAP1 is a known scaffold in MAPK signaling. IQGAP1 directly binds Raf^37^, MEK^44^, and ERK^45^. By assembling signaling complexes, IQGAP1 promotes MAPK signaling, where IQGAP1 expression correlates with ERK activation^44,45^. IQGAP3 also associates with at least some MAPK components^10,42^ and may scaffold MAPK. As both Ras activity and IQGAP1 expression level correlate with ERK activation, it has been proposed that IQGAPs may alter Ras activation^18,19,23^, which would contribute to changes in ERK activity. To determine if IQGAPs modulate Ras, we compared Ras activation in cells with knockdown of IQGAP1 or IQGAP3 to those with normal IQGAP levels. EGF stimulated a rapid increase in active Ras and a subsequent return to basal levels; the amounts of active Ras were similar at all time points regardless of the presence of IQGAP1 or IQGAP3. In contrast to our data, there is one previous publication which reported that stable knockdown of IQGAP3, but not of IQGAP1, reduced levels of active Ras^19^. Several factors may contribute to the different findings between the studies. We used serum starved HeLa cells with transient knockdown of IQGAP1 or IQGAP3 and examined the amount of active Ras in response to EGF. Nojima *et al*.^19^ evaluated basal levels of GTP-bound Ras in normal mouse breast epithelial cells with stable knockdown of IQGAP1 or IQGAP3. Some other observations by Nojima *et al*. also differ from the published literature. Surprisingly, they observed no change in GTP-bound Rac1, Cdc42 or Rho in their knockdown cells. Earlier publications document that knockdown of IQGAP1 led to decreased active Rac1^46,47^ and Cdc42^47^, and increased or decreased (depending on cell type) active RhoA^48,49^. It is possible that the stable knockdown of IQGAP proteins in specific cell clones may lead to cellular responses to maintain homeostasis under these particular conditions which would alter Ras activity. In addition, IQGAP1 can regulate GTPase activity via association with GEFs and GAPs^48,50^, which is another mechanism by which IQGAPs may influence the amount of active GTPases independent of a direct interaction.

Although IQGAP1 and IQGAP3 do not appear to interact directly with Ras, it may be therapeutically useful to target these IQGAP proteins to inhibit Ras oncogenic activities. For example, a cell penetrating peptide corresponding to the WW domain of IQGAP1 inhibited Ras- and Raf-driven tumorigenesis in cultured cells and mice^51^. Knockdown of IQGAP3 by siRNA in a mouse model of metastatic lung cancer reduced tumorigenesis and attenuated EGF-induced ERK phosphorylation^10^. As mentioned in the previous paragraph, IQGAP1 binds directly to and scaffolds several components of the MAPK cascade^37,44,45^ and IQGAP3 associates with MAPK components^10,42^. Therefore, targeting the IQGAP proteins may be effective in Ras-mediated tumors by attenuating MAPK signaling rather than attenuating Ras.

Identifying and correcting errors is essential to science^52^. Consistent with this idea, lack of reproducibility is one of the most pressing problems facing experimental science^53–56^. Under stringent, carefully controlled and physiologically-relevant conditions we failed to observe an interaction between Ras and IQGAP1 or IQGAP3. Our results emphasize the importance of protein concentrations when attempting to determine relevant endogenous protein-protein interactions. Associations that are not robust enough to be detected at physiological concentrations require careful experimentation to ascertain possible biological relevance. The notion that IQGAP1 and/or IQGAP3 are direct effectors of Ras has been promulgated in recent literature^20–26^ and cited in US patents^27^. It is therefore particularly important that we communicate our negative results to prevent researchers from using their valuable time, energy, and resources in attempting to expand upon this interaction. Our data introduce a cautionary reflection for targeting the interaction of Ras with IQGAP for the development of novel therapeutic agents.

## Methods

### Materials

HEK293, HeLa, and A549 cells were obtained from American Type Culture Collection. All reagents for tissue culture were from Life Technologies. Protein A-Sepharose, glutathione-Sepharose, and calmodulin-Sepharose 4B were purchased from GE Healthcare. PVDF membranes were purchased from Millipore Corp. Anti-IQGAP1 polyclonal antibodies have been characterized previously^57^. The anti-calmodulin monoclonal antibody has been characterized previously^58^. Anti-IQGAP1 clone AF4 monoclonal antibody (catalog no. 05–504) was purchased from EMD Millipore. Anti-IQGAP3 rabbit polyclonal antibody was produced by Cocalico Biologicals as described below. Anti-C-Raf antibody (catalog no. 94422 S), anti-HSP90 antibody (catalog no. 4877 S), anti-phospho-ERK1/2 antibody (catalog no.4377 S) and anti-ERK1/2 antibody (catalog no. 9107 S) were obtained from Cell Signaling. Anti-Cdc42 antibody (catalog no. sc-8401), anti-GFP antibody (catalog no. sc-9996), IQGAP1 siRNA (catalog no. sc-35700), and IQGAP3 siRNA (catalog no. sc-78744) were obtained from Santa Cruz Biotechnology. Silencer Negative Control siRNA No. 1 (catalog no. AM4611) and non-immune rabbit serum (catalog no. 10510) were obtained from Thermo Fisher Scientific. Anti-tubulin antibody (catalog no. T5201), anti-IQGAP3 mouse polyclonal antibody (catalog no. SAB1401986-50UG), anti-Ras clone RAS10 mouse monoclonal antibody (catalog no. 05–516), anti-FLAG M2 antibody (catalog no. F3165), anti-FLAG M2 magnetic beads (catalog no. M8823), and recombinant epidermal growth factor (EGF) (catalog no. srp3027) were obtained from Sigma. InstantBlue protein stain was purchased from Expedeon. G-Lisa Ras Activation Assay (catalog no. BK131) kit was purchased from Cytoskeleton. Blocking buffer and infrared dye-conjugated (IRDye) antibodies, both anti-mouse and anti-rabbit, were obtained from LI-COR Biosciences. All restriction enzymes were from New England Biolabs.

### Generation of anti-IQGAP3 rabbit polyclonal antibody

*E. coli* were transformed with pGEX-4T-IQGAP3N and induced with Isopropyl β-D-1-thiogalactopyranoside (IPTG). GST-IQGAP3N protein (amino acids 2–697) was purified with glutathione-Sepharose, eluted with PBS containing 10 mM glutathione, and dialyzed against PBS with 6 mM 2-mercaptoethanol. The protein was sent to Cocalico Biologicals, Inc. who produced anti-IQGAP3 antiserum from rabbits injected with the protein.

### Plasmid construction and expression

The construction of Myc-tagged and GFP-tagged IQGAP1 have been previously described^59^. GFP-IQGAP2 was a gift from Wadie Bahou^8^. The IQGAP3 DNA sequence was derived from a commercially-available human IQGAP3 cDNA (Dharmacon). To generate pcDNA3-Myc-IQGAP3, full length IQGAP3 (corresponding to amino acids 2–1631) was amplified and inserted into pcDNA3 vector (Thermo Fisher Scientific) at BamHI-XbaI site in two steps. First IQGAP3 was amplified. The PCR products and vector were cut with BamHI and XbaI and the C-half of IQGAP3 was ligated into pcDNA3, making pcDNA3-Myc-IQGAP3-C. Next IQGAP3 was amplified again and PCR was used to generated a second BamHI site. The vector and PCR products were digested with BamHI and the N-half of IQGAP3 was inserted into pcDNA3-Myc-IQGAP3-C to create pcDNA3-Myc-IQGAP3. pGEX-2T-IQGAP3 was generated in two steps. First, full length IQGAP3 was amplified from cDNA, cut with BamHI and EcoRI to create a C-half fragment, and inserted into pGEX-2T at the BamHI-EcoRI site to make pGex-2T-IQGAP3C. Second, IQGAP3 was amplified again, digested with BamHI, and inserted into pGEX-2T-IQGAP3C at the BamHI site, producing pGEX-2T-IQGAP3. For GFP-IQGAP3, the full length IQGAP3 sequence was amplified and inserted into vector pEGFP-C1 (Clontech) at the BamHI-EcoRI site in two steps. First, the N-half of IQGAP3 was amplified from cDNA, digested with BamHI and inserted into pEGFP-C1 at BglII site, making pEGFP-IQGAP3N. Second, the C-half of IQGAP3 was digested from pGEX-2T-IQGAP3 with XbaI and EcoRI and inserted into pEGFP-IQGAP3N at the XbaI-EcoRI site making full length pEGFP-IQGAP3. To generate GST-IQGAP3N, the IQGAP3 fragment corresponding to amino acids 2–697 was amplified by PCR from cDNA. pGEX-4T (GE Healthcare) and the IQGAP3 fragment were digested with EcoRI and XhoI and the IQGAP3 fragment was inserted at the EcoRI-XhoI site. GST-tagged pGEX-2T-Cdc42-Q61L was a gift from Darerca Owen^60^. pRK-myc-Cdc42-wt (Addgene plasmid #12972) and pRK-myc-Cdc42-T17N (Addgene plasmid #12973) were a gift from Gary Bokoch. To construct pcDNA3-Myc-Cdc42-Q61L, Cdc42-Q61L was cut from pGEX-2T-Cdc42-Q61L with EcoRI. pcDNA3 vector was cut with XbaI. Blunt ends were generated with T4 polymerase on both the vector and the Cdc42-Q61L fragment. Vector and fragment were both then digested with BamHI and then the Cdc42-Q61L sequence was inserted. To construct pcDNA-Myc-C-Raf, C-Raf was amplified from plasmid Flag-Raf-1-GFP and digested with BamHI. pcDNA3 vector was digested with XbaI. Blunt ends were generated with T4 polymerase then cut with BamHI and C-Raf was inserted into pCDNA3 vector at BamHI site. The Ras constructs were generated as follows. The sequence for H-Ras G12V was amplified from pBABE-HRAS-G12V (a gift from Joan Brugge), K-Ras G12V was amplified from pBABE-puro-KRAS-V12 (a gift from William Hahn, Addgene plasmid #9052), and N-RAS G12V was amplified from pLenti-PGK-NRAS(G12V) (a gift from Daniel Haber, Addgene plasmid 35632). For FLAG-tagged Ras constructs, the amplified Ras sequences were cloned into pCMV-(DYKDDDDK)-N (Clontech) vector by standard cloning techniques. To construct GST-tagged Ras, the amplified Ras sequences were cloned into pGEX-2T vector by standard cloning techniques. The sequences of all plasmids were confirmed by Sanger sequencing. All constructs migrated to the expected position in SDS-PAGE.

### Preparation of fusion proteins

GST-tagged H-Ras, K-Ras, N-Ras, and Cdc42 proteins were expressed in *Escherichia coli*. Bacteria were lysed by sonication in PBS supplemented with 0.2 mM phenylmethylsulfonyl fluoride and 10 mM dithiothreitol. Triton X-100 was added to 1% (v/v) and debris removed by centrifugation. Samples were loaded on glutathione-Sepharose columns and washed with PBS containing 10 mM dithiothreitol.

### Cell culture and transfection

HEK293, A549, and HeLa cells were grown in Dulbecco’s Modified Eagle Medium (Thermo Fisher Scientific) with 10% fetal bovine serum (FBS). All DNA transfections were performed with 2 µg of plasmid using Lipofectamine 2000 (Thermo Fisher Scientific) prepared in 500 µL of Optimem (Thermo Fisher Scientific) following the manufacturer’s instructions. siRNA transfections were performed with 3 µL of siRNA reconstituted following the manufacturer’s instructions using RNAiMAX (Thermo Fisher Scientific) prepared in 500 µL of Optimem.

### Western blotting

After transfer, PVDF membranes were incubated in blocking buffer for 1 h at 22 C. Membranes were then incubated in primary antibody overnight at 4 C, followed by incubation with infrared-dye-conjugated secondary antibodies for 1 h at 22 C. All primary antibodies were diluted 1:1000, except the anti-IQGAP1 polyclonal antibody and the anti-tubulin antibody, which were diluted 1:2000 and 1:10,000, respectively. All secondary antibodies were diluted 1:10,000. For Western blotting calmodulin, proteins were fixed to the membrane with 0.2% glutaraldehyde for 45 min at 22 C before the blocking step, essentially as previously described^57^. Antigen-antibody complexes were detected using an Odyssey imaging system (Li-Cor Biosciences). Bands were quantified with Image Studio 2.0 (Li-Cor Biosciences).

### Immunoprecipitation

HEK293 cells were grown to confluence, washed with ice cold PBS (Corning), and lysed by sonication for 10 s in lysis buffer buffer (150 mM NaCl, 1% Triton X-100, 200 mM Tris-HCl, pH 7.4) containing Halt Protease and Phosphatase Inhibitor (Thermo Fisher Scientific). Lysates were pre-cleared with Protein A-Sepharose for 1 h at 4 C and then incubated with anti-IQGAP3 or anti-IQGAP1 rabbit polyclonal antibody and Protein A-Sepharose for 4 h at 4 C. Samples were washed 5 times with lysis buffer, then resolved by SDS-PAGE, transferred to PVDF, and analyzed by Western blotting. To evaluate binding between IQGAP3 and Cdc42, subconfluent cells were transfected with Myc-IQGAP3 alone, Myc-IQGAP3 plus Myc-Cdc42-wt, Myc-IQGAP3 plus Myc-Cdc42-Q61L, or Myc-IQGAP3 plus Myc-Cdc42-T17N and allowed to grow to confluence prior to lysis.

### FLAG-Ras immunoprecipitation

One subconfluent 10-cm dish each of HEK293 cells was transfected with Flag- tagged H-, K-, or N-Ras or Flag vector alone and allowed to reach confluence. Cells were washed with ice-cold PBS and lysed in lysis buffer by sonication for 10 s. Insoluble material was precipitated by centrifugation at 20,000 X g for 10 min at 4 C. After pre-clearing with glutathione-Sepharose for 1 h at 4 C, supernatants were incubated with anti-FLAG M2 Magnetic Beads for 4 h at 4 C. Beads were washed 5 times with lysis buffer and bound proteins were resolved by SDS-PAGE, transferred to PVDF, and analyzed by Western blotting.

### GST pull down

Subconfluent 10-cm dishes of HEK293 cells were transfected with Myc-C-Raf alone, both Myc-C-Raf and Myc-IQGAP1, or Myc-C-Raf and Myc-IQGAP3, and allowed to grow to confluence. Cells were lysed by sonication for 10 s in 1 mL of lysis buffer supplemented with 1 mM EGTA. Lysates were pre-cleared with glutathione-Sepharose for 1 h at 4 C. Equal amounts of lysate were then incubated with GST-tagged H-, K-, or N-Ras, GST-tagged Cdc42, or GST alone attached to glutathione-Sepharose beads for 3 h at 4 C. Beads were washed 5 times with lysis buffer and bound proteins were resolved by SDS-PAGE. Gels were cut around 60 kDa and high molecular weight proteins were transferred to PVDF and analyzed by Western blotting. Proteins in the lower half of the gel were visualized by soaking the gel in InstantBlue protein stain for 30 min at 22 C.

### Calmodulin pull down

Confluent HEK293 cells were lysed by sonication for 10 s in lysis buffer supplemented with either 1 mM CaCl_2_ or 1 mM EGTA. Equal amounts of lysate were pre-cleared with glutathione-Sepharose for 1 h at 4 C, then incubated with calmodulin-Sepharose beads or glutathione-Sepharose control beads for 90 min at 4 C. Beads were washed 5 times with lysis buffer and bound proteins were analyzed by SDS-PAGE and Western blotting.

### Ras G-Lisa activation assay

The Ras kinetic assay was performed with G-Lisa Ras Activation kit (Cytoskeleton) according to the manufacturer’s instructions. Briefly, HeLa cells were transfected with Control, IQGAP1, or IQGAP3 siRNA for 72 h in biological triplicate. After overnight serum starvation, cells were stimulated with 100 ng/mL of EGF for 0, 2, 5, or 10 min. Cells were washed with ice cold PBS, lysed, and the G-Lisa assay was performed. Briefly, equal amounts of protein were incubated in the wells of the G-Lisa 96-well plate and then the plate was washed. Recombinant Ras binding domains that are linked to the surface of a 96-well plate bind only GTP-Ras and the amount bound is quantified via an enzyme-linked antibody and colorimetric substrate, which absorbs light at 490 nm. Absorbance readings were performed with a Biotek Instruments Synergy 4 plate reader. Mean and standard error were plotted with Graph Pad Prism software and statistical significance was determined by two-sided Student’s t-test. An aliquot of cell lysate processed in parallel was analyzed by SDS-PAGE and Western blotting to evaluate the amounts of IQGAP1 and IQGAP3 present.

### ERK activation

HeLa cells were transfected with Control, IQGAP1, or IQGAP3 siRNA for 72 h. After overnight serum starvation, cells were stimulated with 100 ng/mL of EGF for 0, 2, 5, or 10 min. Cells were washed with ice cold PBS, lysed in lysis buffer and equal amounts of protein were examined by SDS-PAGE and Western blotting and probed with antibodies to phospho-ERK1/2, total ERK, IQGAP1 and IQGAP3.

## Supplementary information

Dataset 1
