# Supplementary material for: Endogenous IQGAP1 and IQGAP3 do not functionally interact with Ras
Source: Sci Rep. 2019 Jul 30;9:11057. doi: 10.1038/s41598-019-46677-9 (PMC6667474; doi:10.1038/s41598-019-46677-9)

Supplementary Figure Panels

Manuscript Title: Endogenous IQGAP1 and IQGAP3 do not functionally interact with Ras

Authors: Chase J. Morgan, Andrew C. Hedman, Zhigang Li, David B. Sacks*

From the Department of Laboratory Medicine, National Institutes of Health, 10 Center Drive, Bethesda, Maryland 20892, USA.

*To whom correspondence should be addressed: David B. Sacks, Department of Laboratory Medicine, National Institutes of Health, 10 Center Drive, Bethesda, Maryland 20892, USA. sacksdb@nih.gov

Supplementary Figure 1.
Full-length blots and gels

1.A

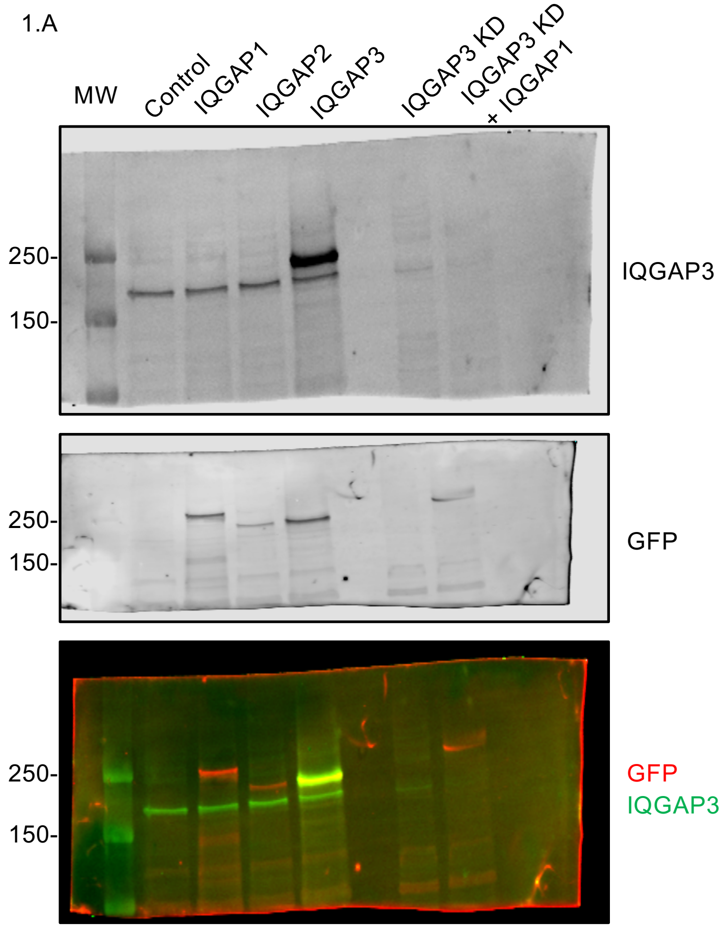

1.B

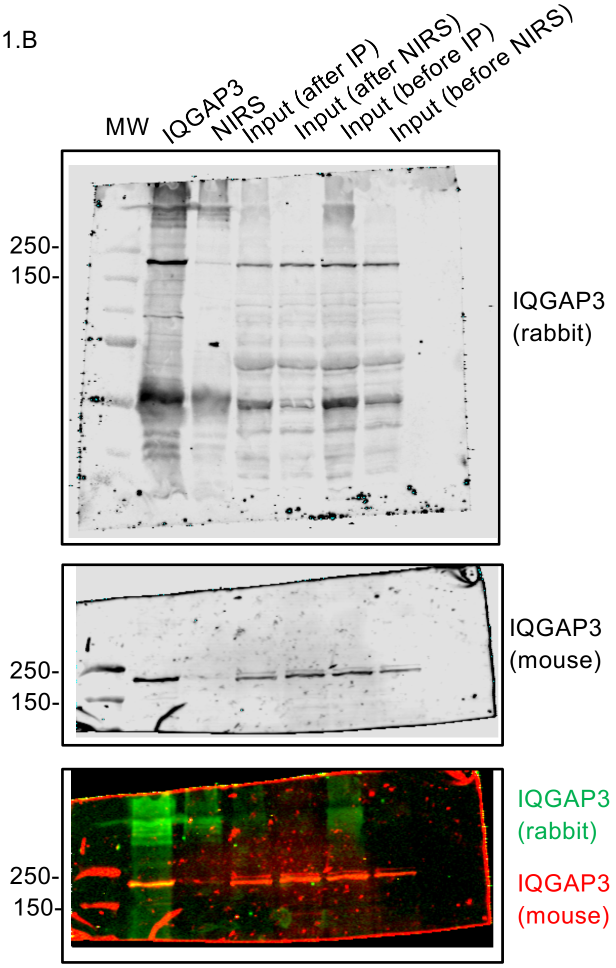

1.C

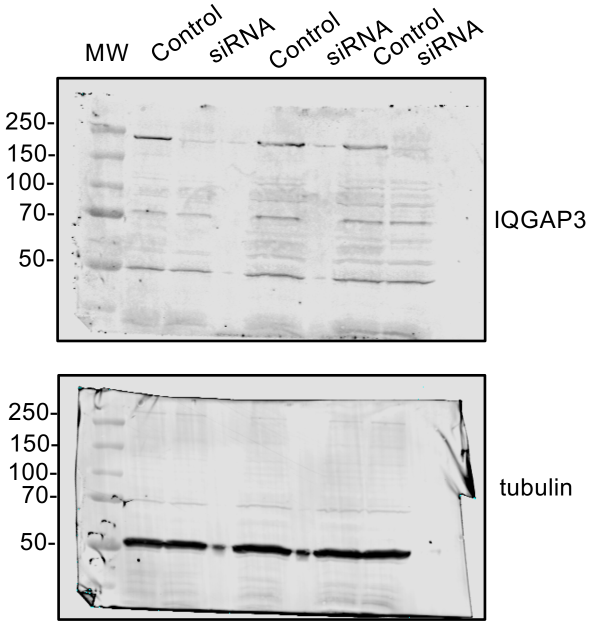

Supplementary Figure 2.

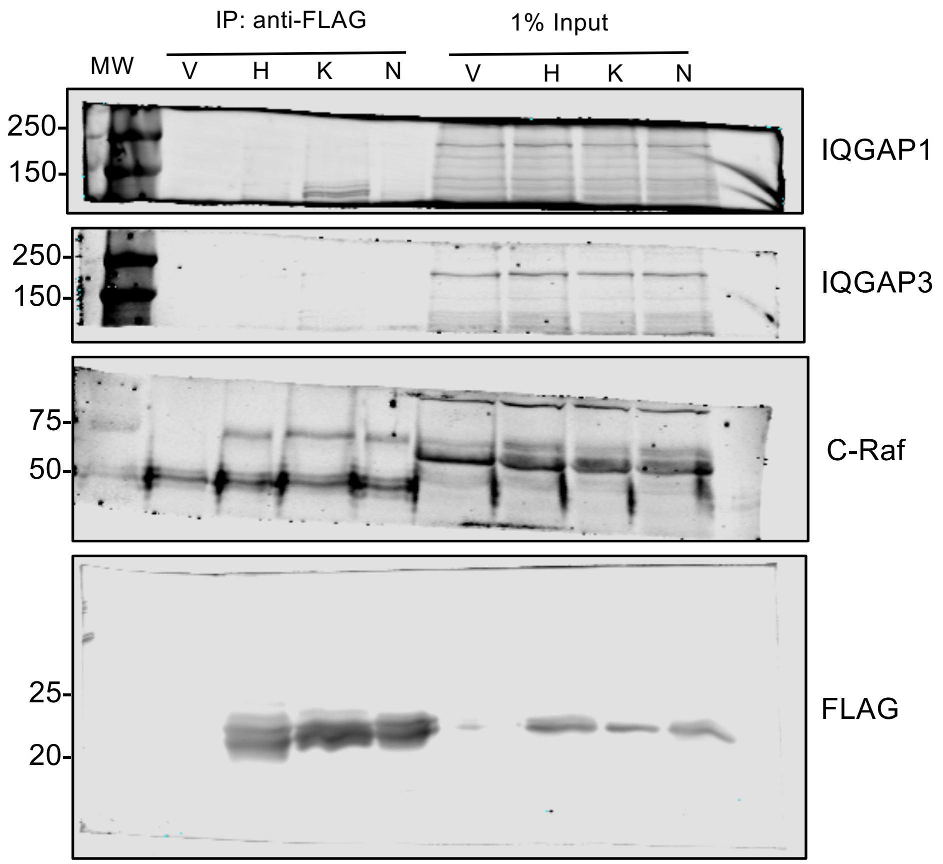

Supplementary Figure 3.

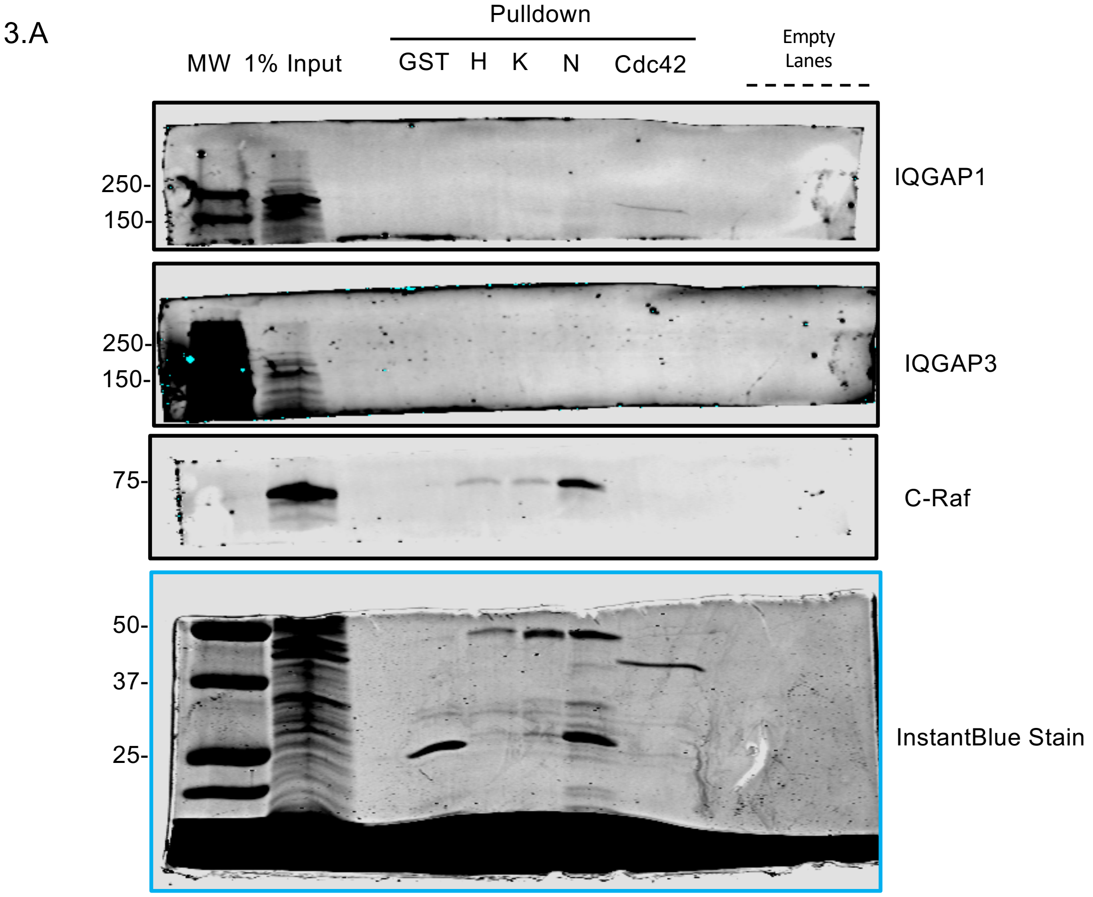

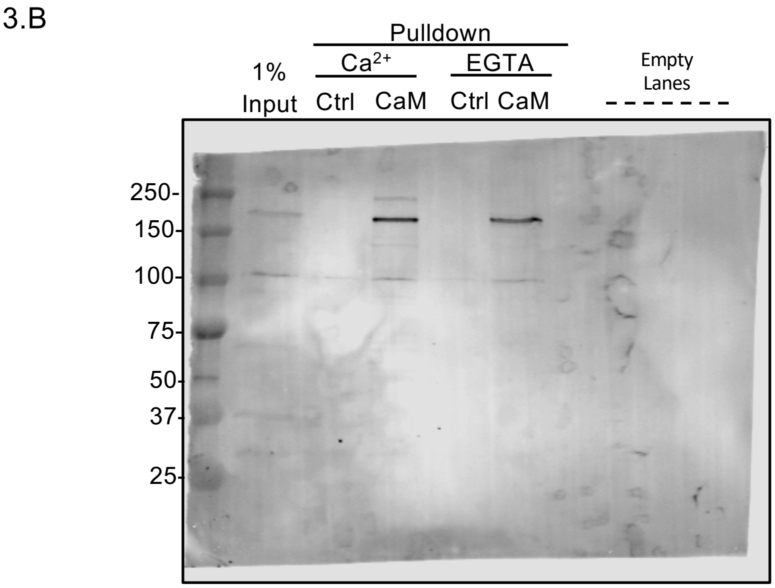

Supplementary Figure 4.

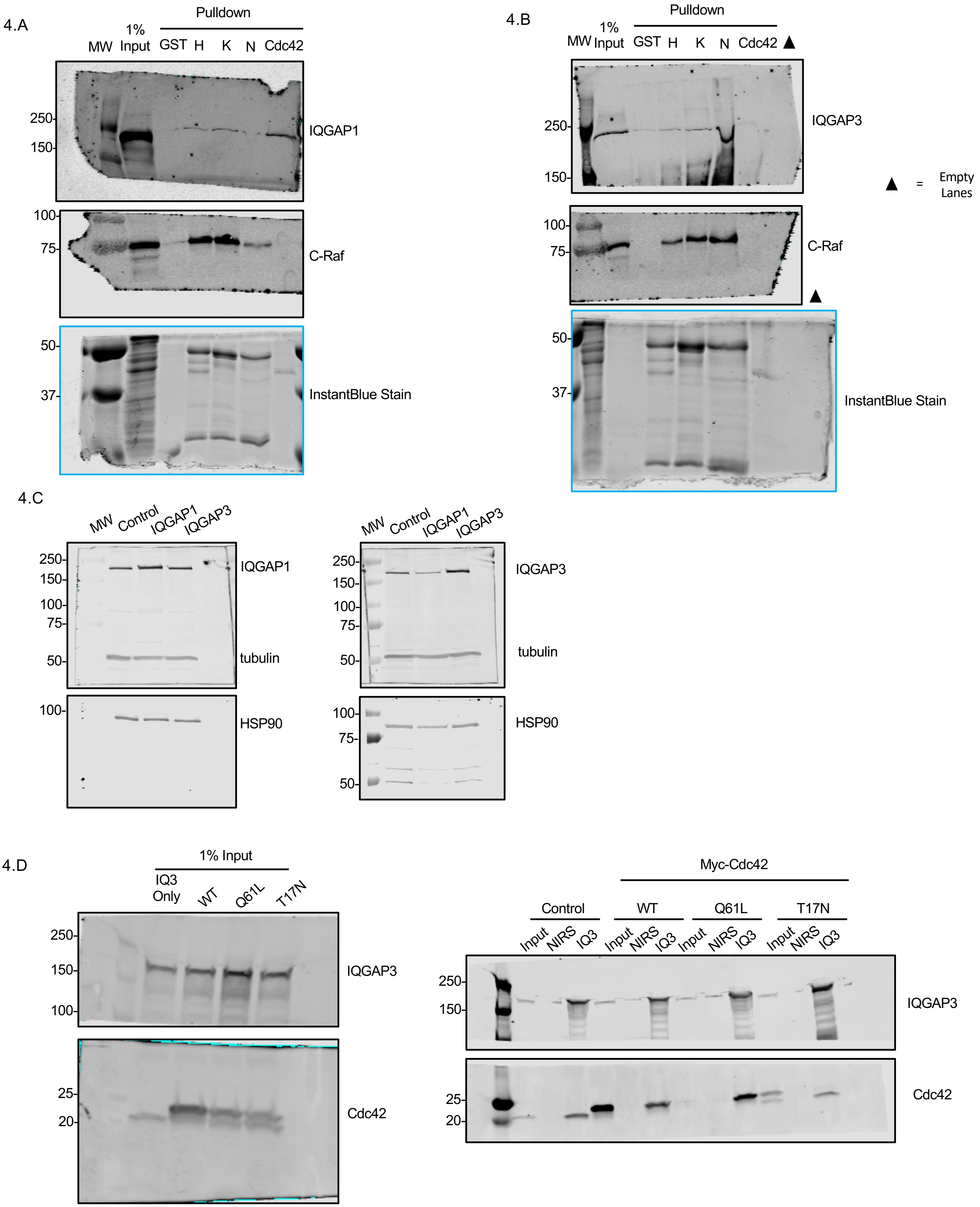

Supplementary Figure 5.

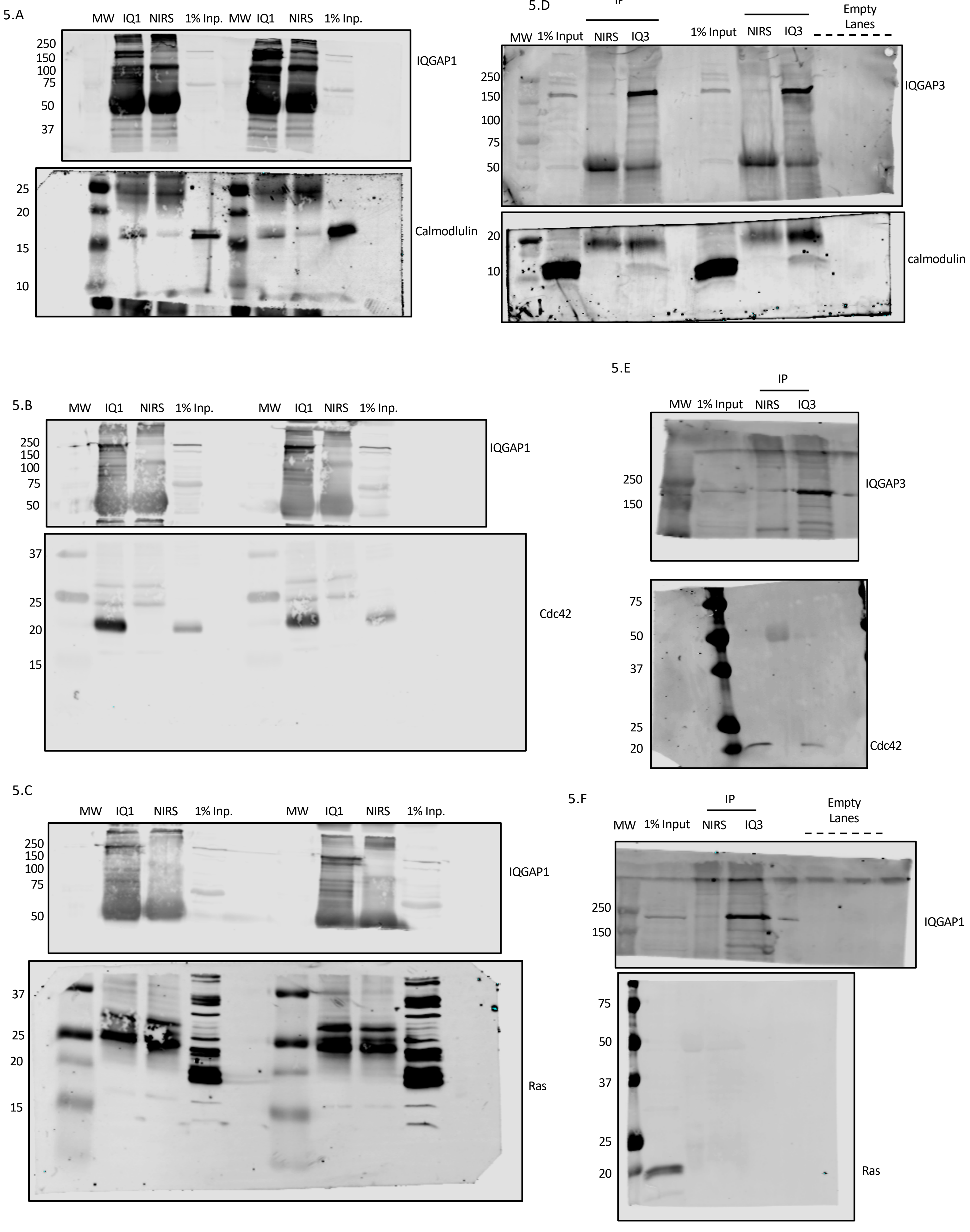

Supplementary Figure 6.

6.B

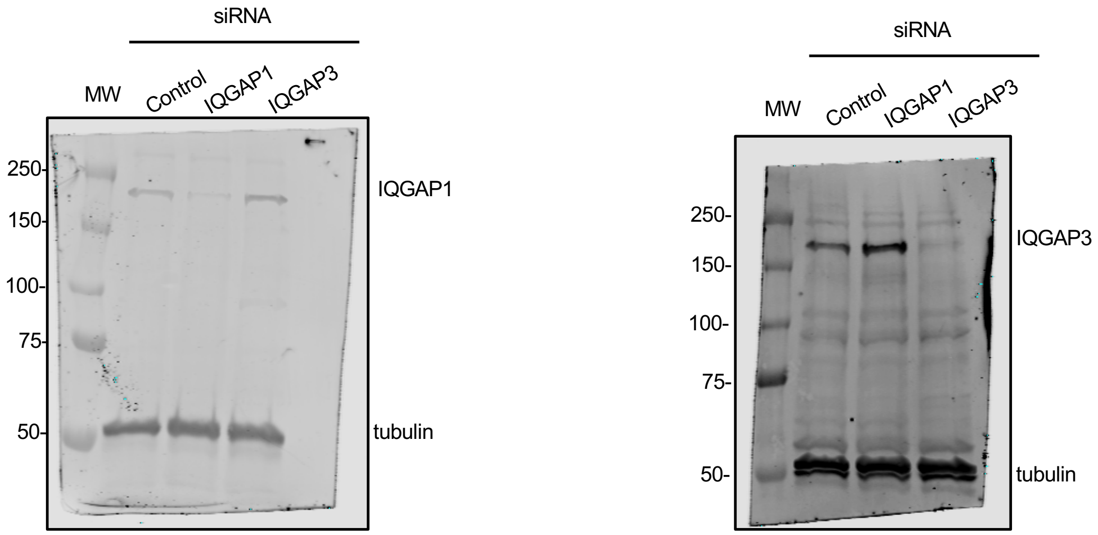

6.C

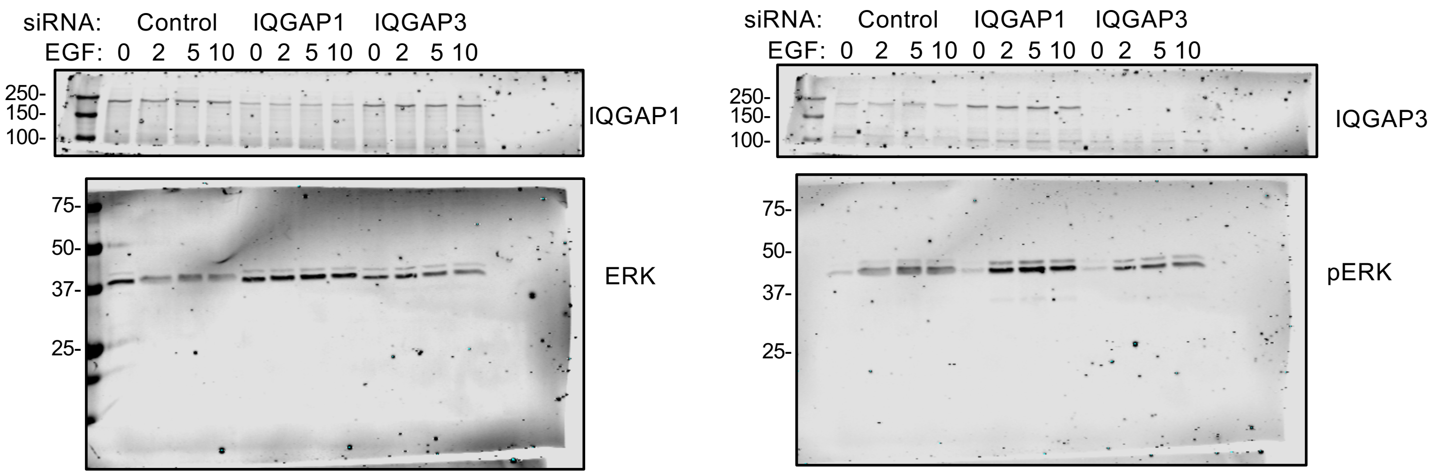

Supplement: Supplementary file 1 — Dataset 1 [file 41598_2019_46677_MOESM1_ESM.pdf]
